# Prevalence and predictors of malnutrition during adolescent pregnancy in southern Ethiopia: a community-based study

**DOI:** 10.1186/s12884-022-04460-1

**Published:** 2022-02-16

**Authors:** Belete Yimer, Awraris Wolde

**Affiliations:** 1 Department of Human Nutrition, Debre Markos University, Debre Markos, Ethiopia; 2 Department of Public Health, Debre Markos University, Debre Markos, Ethiopia

**Keywords:** Adolescent, Pregnancy, Malnutrition, MUAC

## Abstract

**Background:**

Adolescent pregnancy is a major public health problem with significant medical, nutritional, social and economic risk for mothers and their infants. The purpose of this study was to determine prevalence and predictors of malnutrition among pregnant adolescents in Kore district, southern Ethiopia.

**Methods:**

Data were obtained from randomly selected consenting four hundred twenty five pregnant adolescents on March 2018 using interviewer-administered questionnaire and mid upper arm circumference (MUAC) measurement. A multivariable logistic regression analysis was used to identify the predictors of malnutrition in adolescent pregnancy.

**Results:**

The study showed that 26.4% of study participants were malnourished (MUAC < 22 cm). Not owning livestock (AOR = 1.67, 95% CI = 1.26–2.19), unintended pregnancy (AOR = 1.36, 95% CI = 1.08–1.65), excess physical work in pregnancy (AOR = 1.29, 95% CI = 1.02–1.62) and being in the second (AOR = 1.70, 95% CI = 1.09–2.65) or third (AOR = 1.99, 95% CI = 1.29–3.07) pregnancy trimester were positively associated with malnutrition risk. Improved dietary intake in pregnancy (AOR = 0.46, 95% CI = 0.33–0.63) and support perceived by adolescents in pregnancy (AOR = 0.59, 95% CI = 0.43–0.82) were negatively associated with malnutrition risk.

**Conclusion:**

More than one-quarter of the study population were malnourished. The information provides insight into the public health strategies to reduce malnutrition risk of the pregnant adolescents. Interventions aimed at improving socioeconomic status, dietary practice and physical work/activity through effective supports in pregnancy are recommended.

## Background

Malnutrition is excess or deficiency state of nutrition in the body. It commonly applies to the latter form of malnutrition in the context of low- and middle- income countries (LMICs). Maternal malnutrition is generally common among women in LMICs and particularly high among those in south Asia and Africa regions affecting a quarter to a third of women [1, 2]. The Ethiopian Demographic Health Survey (EDHS) 2016 reported that 22% of non-pregnant and non-postpartum Ethiopian women were chronically malnourished [3].

Importantly, in many LMICs of the world, child-bearing early in adolescence due to early marriage is common with significant medical, nutritional, social and economic risk [4–6]. In Ethiopia, 13% of women aged 15–19 years have begun childbearing as of Ethiopian DHS 2016. The percentage of adolescents who have given birth or are pregnant with their first child has decreased since 2000, from 16 to 13%. Adolescents (15%) in rural areas are three times more likely to have begun childbearing than 5% of their urban peers [3]. It has been argued that the real risks of early childbearing are concentrated in the poverty and the environmental and social circumstances, rather than the biologic vulnerability, of young mothers [7–9].

Previous studies in LMICs that directly compared nutritional status of pregnant adolescents versus pregnant adult women are limited. A study from Malawi found that adolescent mothers were more likely to be malnourished than older mothers [10]. Adolescents’ nutrition and weight status and lifestyle practices prior to and during pregnancy profoundly influence pregnancy and birth outcome [11–13]. Despite this, in Ethiopia, data on malnutrition during pregnancy is scarce; almost non-existent for pregnant adolescent mothers [14]. Thus, the purpose of this study was to determine prevalence and predictors of malnutrition in pregnant adolescents living in a rural area.

## Methods

### Study site, design and population

A community-based cross-sectional study was conducted among pregnant adolescents in Kore district, southern Ethiopia on March 2018. The district is divided into 21 rural and 4 urban kebeles (smallest administrative unit) and its estimated population is 156,035, of which, more than 95% live in the rural kebeles. The food production system in the area is characterized by mixed-crops and livestock production. Barley is among the major food crops produced in the area. Most of the communities in Ethiopia including the one in the study area, are characterized by patriarchal norms and high gender disparity. The study was concerned to the issues faced by pregnant adolescents aged 15–19 years living in a rural area in relation to malnutrition.

### Sample size estimation and population selection

The sample size was estimated by assuming a 50% malnutrition, a 95% confidence interval, a 5% margin of error, and a 10% non-response rate and this yielded a sample size of 422. The selection of the study subjects was occurred on two stages -cluster and individual. To select the kebeles (cluster level), the list of all rural kebeles in the district was considered as a sampling frame and ten kebeles were randomly selected. The individual level was involved selecting all pregnant adolescents within the selected kebeles. Following the selection of clusters, pregnant adolescents were identified by self-report, from the records of health extension workers (HEWs) providing a community based maternal and child services in each kebele and urine testing when pregnancy was doubtful. Pregnant women were identified from the report of health extension workers in the kebeles Total of 436 pregnant adolescents were identified from the selected kebeles and all of them were recruited recognizing that little difference from the sample size estimate.

### Data collection and measurement processes

Data were collected using interviewer-administered questionnaire and mid upper arm circumference (MUAC) measurement. Initially questionnaire was written in English and translated to the local languages then back translated to English. The questionnaire include information on socio-demographic and-economic characteristics, dietary and lifestyle/work related practices, and obstetric and healthcare history.

Improved eating habits during pregnancy was assessed using the items adapted from the national nutrition survey conducted by the Ethiopian Health and Nutrition Institute in 2009. Pregnant adolescents who responded affirmatively to at least either of the two items asking whether they had (1) increased their meal amount and (2) increased their meal frequency were classified as having improved dietary practices during pregnancy. Pregnant adolescents were asked about received support during pregnancy: (1) material aid (2) assistance with tasks (3) advice or information and (4) listening while one expresses beliefs or feelings [15]. For example, to assess task support, pregnant adolescents were asked, “In this pregnancy, did you get help from anyone with things you had to do such as errands, household tasks, or childcare?” Responses were recorded as yes or no. Then, a value of 1 was assigned if the subjects answered yes to any of the support items and 0 otherwise. The values were summed to produce a perceived support scores. The scores were dichotomized as received support for pregnant adolescents having a value of 1 and above and did not receive for those who had a value of 0.

We use the definition of malnutrition during adolescent pregnancy in this study to refer to MUAC of pregnant adolescents below 22 cm [16]. A MUAC of each adolescent mother’s left arm at the mid-point between the tips of the shoulder (olecranon process) and the elbow (acromion process) was measured using inelastic insertion type tape. Measurement of MUAC was made while the woman stood-up relaxed with her hand hanging down. After checking that the tape was applied with correct tension (neither too loose nor too tight), the MUAC of the woman was read and recorded to the nearest 0.1 cm. MUAC measurement was taken with a pair of data collectors following the recommended measurement technique [17].

The data were collected by trained female data collectors. A 2 day intensive training prior to the pre-test and an additional day of training was given with the final version of the questionnaire before the actual interviews. Trained supervisors and principal investigators conducted field supervision. All completed questionnaires were collected by respective supervisors and checked overnight and doubtful interviews and MUAC measurement were repeated.

### Statistical analysis

Data were entered and validated using Epidata version 3.1. STATA version 14 was used for data analysis. In this study the dependent variable malnutrition was coded as 1 if the pregnant adolescents were MUAC < 22 cm and, 0 otherwise. First, bivariate analyses were carried out. Proportions were compared by malnutrition using Chi-square tests. The independent variables were examined for their association with malnutrition using multivariable logistic regression. Variables that showed a significant association with malnutrition in the bivariate analyses were entered in a multivariable logistic model. All tests were two-sided and a *p*-value of < 0.05 was used as a cut-off point to declare statistical significance. We present the results as means ± SD, output of the logistic regression as odds ratios (OR) with 95% confidence intervals.

## Results

### Characteristics of respondents and prevalence of malnutrition

The basic characteristics of the study participants are given in Table 1. A total of 425 (97%) pregnant adolescents were included in the analysis. Their mean age was 17.1 (SD ±1.2) years. Two-thirds (66.1%) of the study participants were in 15–17 years age group and 68.2% of them were illiterate. Nearly half (48.8%) of the study participants were in the third trimester of their pregnancy and more than half (51.1%) of them were primigravidae (Table 1).

**Table 1:** Socio-demographic, prenatal health and dietary related characteristics of malnutrition of pregnant adolescents in Kore district, southern Ethiopia, 2018

Variable	Total (%)	Normal (%)	Malnourished (%)	*P*-value
Age groups				0. 10
15–17 years	281 (66.1)	207 (73.7)	74 (26.3)	
18–19 years	144 (33.9)	105 (72.9)	39 (27.1)	
Educational status				0.21
None	290 (68.2)	212 (73.1)	78 (26.9)	
Primary	106 (25.0)	85 (80.3)	21 (19.7)	
Secondary	29 (6.9)	21 (73.7)	8 (26.3)	
Drinking water source				< 0.001
Not improved	183 (43.0)	126 (69.1)	57 (30.9)	
Improved	242 (57.0)	187 (77.4)	55 (22.6)	
Own any livestock				0.01
Yes	273 (64.3)	208 (76.2)	65 (23.8)	
No	152 (35.7)	106 (70.0)	46 (30.0)	
Own any farm land				0.08
No	158 (37.2)	116 (73.2)	42 (26.8)	
Yes	267 (62.8)	194 (72.6)	73 (27.4)	
Previous pregnancies				0.28
0	217 (51.1)	154 (70.8)	63 (29.2)	
1	140 (33.0)	103 (73.5)	37 (26.5)	
≥ 2	68 (15.9)	51 (74.6)	17 (25.4)	
Pregnancy intention				0.02
Intended	291 (68.5)	217 (74.6)	74 (25.4)	
Not intended	134 (31.5)	92 (68.5)	42 (31.5)	
Pregnancy trimesters				0.01
First	60 (14.0)	49 (82.3)	11 (17.7)	
Second	158 (37.2)	117 (74.3)	41 (25.7)	
Third	207 (48.8)	146 (70.7)	61 (29.3)	
Antenatal visits				0.44
No	109 (25.7)	80 (73.2)	29 (26.8)	
Yes	316 (74.3)	246 (77.7)	70 (22.3)	
Diet practice in pregnancy				0.004
Not improved	201 (47.4)	140 (69.6)	61 (30.4)	
Improved	224 (52.6)	173 (77.2)	51 (22.8)	
Workload in pregnancy				0.004
No	278 (65.4)	210 (75.5)	68 (24.5)	
Yes	147 (34.6)	98 (66.9)	49 (33.1)	
Support in pregnancy				0.02
No	309 (72.6)	222 (71.7)	87 (28.3)	
Yes	116 (27.4)	91 (78.7)	25 (21.3)	

The mean MUAC (± SD) of the respondents was 22.3 cm (± 2.8). Of 425 pregnant adolescents studied, 112 had MUAC < 22 cm. This gave a prevalence of 26.4% malnutrition (95% CI: 23.0–29.7%). The detail of malnutrition distribution with the examined characteristics of study participants is given in Table 1.

As Table 1 shows, the higher proportion of malnourished pregnant adolescents were living in the households that did not own any livestock compared to those who owned any livestock (30.0% vs 23.8%; *P* = 0.01). The proportion of malnutrition was 29.3 and 25.7% respectively in pregnant adolescents in the third and second trimesters as compared to 17.7% among those in first trimester (*P* = 0.01). Furthermore, support in pregnancy (*P* = 0.015), dietary practice in pregnancy (*P* = 0.004), workload in pregnancy (*P* = 0.01) and pregnancy intention (*P* = 0.02) were statistically associated with malnutrition. However, women’s age (*P* = 0.10) and their educational status (*P* = 0.21) were not statistically associated with increased risk for malnutrition.

### Predictors of malnutrition among pregnant adolescents

In the bivariate analysis, unprotected drinking water source (COR = 1.54, 95% CI = 1.23–1.85), not owning any livestock (COR = 1.39, 95% CI = 1.08–1.76), unintended pregnancy (COR = 1.34, 95% CI = 1.10–1.57), being in the second (COR = 1.56, 95% CI = 1.02–2.40) or the third (COR = 1.86, 95% CI = 1.22–2.78) pregnancy trimester and excess physical work in pregnancy (COR = 1.54, 95% CI = 1.17–1.76) were positively associated with malnutrition risk. Improved dietary practice in pregnancy (COR = 0.68, 95% CI = 0.55–0.89) and received support in pregnancy (COR = 0.70, 95% CI = 0.53–0.94) were negatively associated with malnutrition risk. However, no differences were observed in malnutrition risk between age groups, educational status, antenatal visits and previous pregnancies (Table 2).

**Table 2:** Predictors of malnutrition in multivariable analysis, Kore district, southern Ethiopia, 2018

Variable	COR (95% CI)	*P*-value	AOR (95% CI)	*P*-value
Age groups				
15–17	0.96 (0.63–1.46)	0.10	0.81 (0.51–1.28)	0.360
18–19	1		1	
Educational status				
None	0.97 (0.80–2.15)	0.28	1.18 (0.67–2.08)	0.56
Primary	0.65 (0.76–2.00)	0.40	1.15 (0.68–1.93)	0.60
Secondary	1		1	
Drinking water source				
Not improved	1.54 (1.23–1.85)	< 0.001	1.20 (0.94–1.53)	0.39
Improved	1		1	
Own any livestock				
Yes	1		1	
No	1.39 (1.08–1.76)	0.01	1.67 (1.26–2.19)	< 0.001
Own any farm land				
Yes	1		1	
No	0.96 (0.51–1.04)	0.08	0.85 (0.58–1.23)	0.39
Previous pregnancies				
0	1		1	
1	0.88 (0.64–1.24)	0.49	0.89 (0.62–1.29)	0.55
≥ 2	0.81 (0.58–1.19)	0.30	0.85 (0.55–1.32)	0.48
Pregnancy intention				
Intended	1		1	
Not intended	1.34 (1.10–1.57)	0.02	1.36 (1.08–1.65)	0.02
Pregnancy trimesters				
First	1		1	
Second	1.56 (1.02–2.40)	0.039	1.70 (1.09–2.65)	0.02
Third	1.86 (1.22–2.78)	0.004	1.99 (1.29–3.07)	0.002
Antenatal visits				
No	1		1	
Yes	1.27 (0.72–2.16)	0.436	1.85 (0.92–3.70)	0.08
Dietary intake				
Not improved	1		1	
Improved	0.68 (0.55–0.89)	0.004	0.46 (0.33–0.63)	< 0.001
Workload in pregnancy				
No	1		1	
Yes	1.54 (1.17–1.76)	< 0.001	1.29 (1.02–1.62)	0.01
Support in pregnancy				
Yes	0.70 (0.53–0.94)	0.02	0.59 (0.43–0.82)	0.001
No	1		1	

*COR* Crude odds ratio, *CI* Confidence interval, *AOR* Adjusted odds ratio

After adjusting for the potential confounding effects of all other variables considered in the multivariable logistic regression model, livestock ownership (AOR = 1.67, 95% CI = 1.26–2.19), pregnancy intention (AOR = 1.36, 95% CI = 1.08–1.65), excess physical work in pregnancy (AOR = 1.29, 95% CI = 1.02–1.62) and pregnancy trimesters were positively associated with malnutrition risk. Improved dietary intake in pregnancy (AOR = 0.46, 95% CI = 0.33–0.63) and had support in pregnancy (AOR = 0.59, 95% CI = 0.43–0.82) were negatively associated with malnutrition risk (Table 2).

However, age (AOR = 0.81, 95% CI = 0.51–1.28), antenatal visits (AOR = 1.85, 95% CI = 0.92–3.70), previous pregnancies and educational status were not showed significant associations with malnutrition risk. In addition, the associations between farm land ownership (AOR = 0.85, 95% CI = 0.58–1.23) and access to improved drinking water source (AOR = 1.20, 95% CI = 0.94–1.53) and malnutrition risk observed in the bivariate analysis were not maintained in the multivariable analysis (Table 2).

## Discussion

This a community-based cross-sectional study reports the prevalence of malnutrition in adolescent pregnancy using MUAC index. The study showed that malnutrition affected more than one-quarter of the study population. In this study not owning livestock, unintended pregnancy, excess physical work in pregnancy and second or third pregnancy trimester were positively associated with malnutrition risk. Whereas improved dietary practice and support perceived by adolescents in pregnancy were negatively associated with malnutrition risk.

The prevalence of malnutrition in our study was almost similar to the 29% reported from the Ethiopian DHS for non-pregnant younger women of reproductive age [3]. However, our finding was higher than the 19.1% reported in previous study among pregnant women from eastern Ethiopia [18]. This difference might be explained by geographical, size and composition and sociocultural characteristics differences in the population of the present and the previous studies.

Because adolescent childbearing is concentrated among teenagers who are poor, and low income youth are at higher risk for nutrition problems, pregnant adolescents may enter pregnancy with reduced nutrient stores and increased risk of nutritional inadequacy. In our study, pregnant adolescents who did not owned livestock were at higher risk for malnutrition. Similar difference in the risk of malnutrition across livestock possession has been described previously [18]. This could be explained by the role of livestock as source of income and /or ensuring better access to animal source foods [19].

As noted, an improved dietary intake was negatively associated with malnutrition risk. This is in line with the finding from earlier similar study [18] where reduced malnutrition risk was reported in pregnant women who improved their dietary practice. The finding in these studies supported the recommendation that pregnant women should improve their dietary practice in pregnancy in order to meet their increased energy and nutrients requirement [20, 21].

Recommendations suggest that pregnant women should increase their average daily energy intake during the second and third trimesters of pregnancy [22]. However, younger adolescents may require higher energy intakes during pregnancy than older women [7]. In this study, pregnancy trimesters were positively associated with malnutrition risk. An increased risk of malnutrition observed in pregnant adolescents in the second or third trimesters could be explained by the unmet increased energy and nutrient needs in these periods [23]. Such finding indicate that special care that should be started earlier in pregnancy to insure that the young mother receives sufficient food for her own increased needs, as well as for the needs of the unborn baby [12].

This study had showed workload in pregnancy was positively associated with malnutrition risk. Other previous study also indicated that women who reported perceived excess physical work during their pregnancy were at increased risk for malnutrition than their counterparts [24, 25]. For pregnant women living in rural setting and earn their livelihood through strenuous agricultural and other labor-intensive activities, such finding is not surprising. This may be explained by unsatisfactory weight gain following the negative energy balance between energy intake and expenditure of pregnant women [26]. These findings indicate that pregnant adolescents should be counselled about adequate rest during pregnancy and advised to reduce strenuous work for themselves and their infants.

Pregnant adolescents can be effective if provided with support. Although support should be valuable to all pregnant mothers, adolescents, who often have few economic resources, and unintended pregnancies, may be especially likely to benefit from effective supports [7] which could provide guidance with respect to proper nutritional practices as well as, needed assistance with physically taxing demands that maybe harmful to pregnant mothers, especially late in pregnancy [27]. Any perceived support received in pregnancy predict reduced risk for malnutrition in our study is consistent with the report in earlier study [28].

In our study, consistent with the findings reported in a previous study done in Ethiopia [18], women’s age and educational status were not showed significant associations with malnutrition risk. However, our findings were inconsistent with some other previous studies [29–31].

There are limitations to this study. Firstly, MUAC measurement cannot be free from errors given multiple data collectors were used. In addition, exposure data obtained via pregnant adolescents self-report creates a potential for reporting biases. Further, despite efforts to involve a wide range of predictors in our study, there still exist some variables uncovered. However, this study has key implications for policy, health service and self-care practice about nutrition issues specific to adolescent pregnancy and as such, promoting a healthy pregnancy outcome for mothers and their infants.

## Conclusion

More than one-quarter of the study population were malnourished; needs priority attention of public health strategies. Livestock ownership, pregnancy intention, workload in pregnancy, pregnancy trimester, dietary practice and support in pregnancy were the predictors of malnutrition during adolescent pregnancy. Interventions aimed at improving socio economic status, dietary practice and physical work through effective supports in pregnancy are recommended. In addition, effective family planning programs could help in preventing unintended pregnancy. Finally, quantitative evidences on energy expenditure and weight gain to supplement the findings of present study on relationship between maternal self-reported workload and malnutrition are needed to further realize the effect of steranous work during adolescent pregnancy in this setting. Qualitative study on opinion of support for pregnant adolescent is recommended in order to add more important information on sociocultural aspects of maternal care during pregnancy.

## Data Availability

All relevant data are within the paper.
